# Comparative transcriptome analysis reveals differences in gene expression in whitefly following individual or combined applications of *Akanthomyces attenuatus* (Zare & Gams) and matrine

**DOI:** 10.1186/s12864-022-09048-9

**Published:** 2022-12-06

**Authors:** Jianhui Wu, Tingfei Sun, Muhammad Hamid Bashir, Baoli Qiu, Xingmin Wang, Shaukat Ali

**Affiliations:** 1 Key Laboratory of Bio-Pesticide Innovation and Application, Engineering Research Centre of Biological Control, South China Agricultural University, Guangzhou, 510642 China; 2 Engineering Research Center of Biological Control, Ministry of Education and Guangdong Province, South China Agricultural University, Guangzhou, 510642 China; 3 Department of Entomology, University of Agriculture, Faisalabad, Pakistan; 4 Chongqing Key Laboratory of Vector Insects, College of Life Sciences, Chongqing Normal University, Chongqing, 401331 China

**Keywords:** *Bemisia tabaci*, *Akanthomyces attenuatus*, Matrine, Synergistic action, Immune response

## Abstract

**Background:**

*Bemisia tabaci* Gennadius (Hemiptera: Aleyrodidae) is a serious pest of crops in different regions of the world. Our recent studies on the joint application of *Akanthomyces attenuatus* (a pathogenic insect fungus) and matrine (a botanical insecticide) against *B. tabaci* have shown promising results. Using RNA sequencing (RNA-Seq), we identified differentially expressed genes involved in whitefly responses to single or mixed applications of *A. attenuatus* and matrine.

**Methods:**

In this study, we compared the transcriptome profiles of *B. tabaci* treated with individual and combined treatments of *A. attenuatus* and matrine to determine variations in gene expression among whiteflies in response to different treatments.

**Results:**

Transcriptomic data analysis showed differential expression of 71, 1194, and 51 genes in response to *A. attenuatus* (BtA), matrine (BtM), and *A. attenuatus* + matrine (BtAM) treatment, respectively. A total of 65 common differentially expressed genes (DEGs) were identified between whiteflies treated with *A. attenuatus* (BtA) and matrine (BtM). A comparison of DEGs across the three treatments (BtA, BtM, and BtAM) revealed two common DEGs. The results also revealed that AMPK signaling, apoptosis, and drug metabolism pathways are likely involved in whitefly defense responses against *A. attenuatus* and matrine infection. Furthermore, a notable suppression of general metabolism and immune response genes was observed in whiteflies treated with *A. attenuatus* + matrine (BtAM) compared to whiteflies treated with individual *A. attenuatus* (BtA) or matrine (BtM) treatments.

**Conclusion:**

Dynamic changes in the number of differentially expressed genes were observed in *B. tabaci* subjected to different treatments (BtA, BtM, and BtAM). To the best of our knowledge, this is the first report on the molecular interactions between whitefly and individual or combined treatments of *A. attenuatus* and matrine. These results will further improve our knowledge of the infection mechanism and complex biochemical processes involved in the synergistic action of *A. attenuatus* and matrine against *B. tabaci*.

**Supplementary Information:**

The online version contains supplementary material available at 10.1186/s12864-022-09048-9.

## Background

The whitefly, *Bemisia tabaci*, is an invasive pest and disease vector with a rapidly increasing global population of over 50 cryptic species known to date [1]. Over the course of the past two decades, the cryptic species *B. tabaci* Middle East-Asia Minor 1 (MEAM1) (formerly known as 'Biotype B') replaced the indigenous *B. tabaci* populations in China [2]. *Bemisia tabaci* MEAM1 can cause direct (via feeding on phloem sap) or indirect damage (by secreting honeydew) to its host plants [2, 3]. *B. tabaci* MEAM1 adults have also been found to transmit over 150 plant viruses to commercial crops [4]. The use of synthetic chemicals for the *B. tabaci* management has resulted in development of insecticide resistance [5]. Furthermore, the heavy application of chemical insecticides has been affecting the natural ecosystem (including air, non-target organisms, and humans as well) which has caused a great awareness about finding sustainable alternates to the synthetic chemical [6]. Recently, the joint use of insect pathogenic fungi and botanical insecticides has emerged as a possible alternative to chemical insecticides [7]. Over the last century, entomopathogenic fungi have been used for the biological control of insect pests [7]. Entomopathogenic fungi are more appealing than other pest control strategies owing to their diversity, metabolite production, and biosafety levels [3]. Among these fungi, *Akanthomyces attenuatus* Zare & Gams is a well-known pathogen of insect pests, including whiteflies, aphids, and thrips [8–12]. However, the field application of *A. attenuatus* and other entomopathogenic fungi has received little attention owing to their slow action [13]. Recently, many studies have investigated the joint application of entomopathogenic fungi with natural enemies, synthetic oils, bacterial metabolite avermectins, and other biopesticides [14–17]. Our recent studies on the combined application of matrine (a botanical insecticide) and entomopathogenic fungi (*A. attenuatus* and *Beauveria brongniartii*) against different insect pests have shown promising results [7, 17, 18].

Innate immunity is the first line of protection against numerous pathogenic illnesses. Conserved signal cascades within the innate immune system against insecticides and natural pathogens have been well documented in whiteflies [19–21]. However, to the best of our knowledge, no detailed investigations have been conducted on the mechanisms responsible for controlling the whitefly immune system when defending against the individual or combined applications of *A. attenuatus* and matrine. The recently available whitefly transcriptome sequences [22, 23], in combination with RNA sequencing (RNA-Seq) technology, a revolutionary tool for measuring levels of gene expression [24, 25], have provided unprecedented opportunities to investigate the transcriptional response of *B. tabaci* to single or mixed applications of *A. attenuatus* and matrine. Using RNA-Seq, we identified differentially expressed genes in *B. tabaci* nymphs treated with single or mixed applications of *A. attenuatus* and matrine and analyzed how the whitefly orchestrates its defense responses to these applications. Altogether, our work provides the first report to reveal the molecular mechanisms of *B. tabaci* responses to single or mixed applications of *A. attenuatus* and matrine and new insights into the whitefly infection process during the synergistic action of the interaction between *A. attenuatus* and matrine.

## Results

### Insect bioassays

**Fig. 1:**
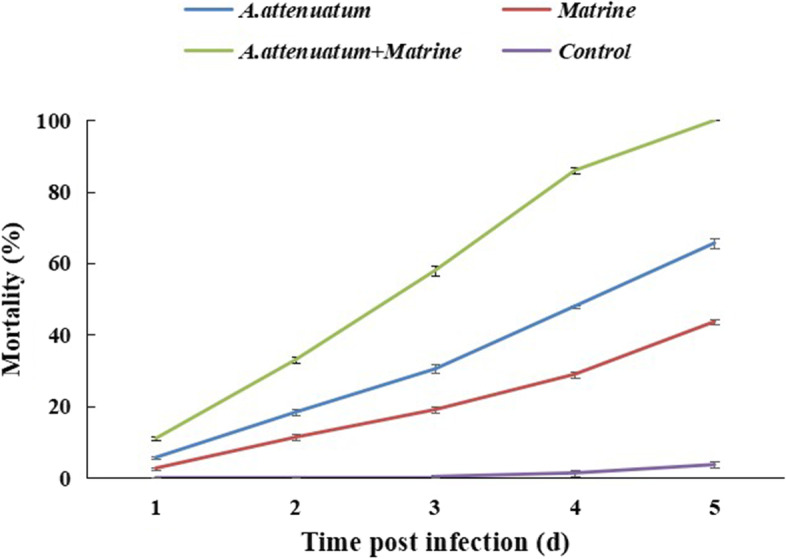
Concentration mortality response of *Bemisia tabaci* to individual or joint treatments of *Akanthomyces attenuatus* and matrine. Error bars indicate standard error of means based on three replicates

The mortality of the 2^nd^ instar *B. tabaci* nymphs treated with *A. attenuatus* and matrine alone or in combination was significantly higher than that of the control. After 3 d of application, *B. tabaci* mortality in response to the combined treatment with *A. attenuatus* and matrine increased substantially compared to the individual treatments. After 5 d of treatment, the mortalities of 2^nd^ instar *B. tabaci* nymphs treated with *A****.*** *attenuatus* (1×10^6^ conidia/mL)*,* matrine (1.0 mg/L), and *A****.*** *attenuatus* + matrine (1×10^6^ conidia/mL + 1.0 mg/L) were 65.67%, 43.67%, and 100%, respectively (Fig. 1).

### Summary of RNA-seq datasets

A total of 659,350,532 raw reads were generated from 12 samples (Additional File 1A). The N reads, low-quality reads, and reads with adapter sequences were removed to ensure the quality and accuracy of sequencing data, and 650,606,120 clean reads were generated that were successfully mapped to 73.3-79.19% of the reference genome; subsequent analysis was based on clean reads. Contigs were obtained first, followed by transcripts, resulting in 35,895 unigenes, of which 23,694 (66.06%) were longer than 1,000 bp. According to the length distribution of *B. tabaci* unigenes, the largest gene length was >1,800 bp (17,742 genes; 49.47 %), the second largest gene length was 201–400 bp (4,835 genes, 13.48 %), and the smallest gene length was 1,601–1,800 bp (1,309 genes, 3.64%) (Additional file 1B). The Pearson’s correlation coefficients showed highly reproducible data across different replicates (Additional file 1C).

**Fig. 2:**
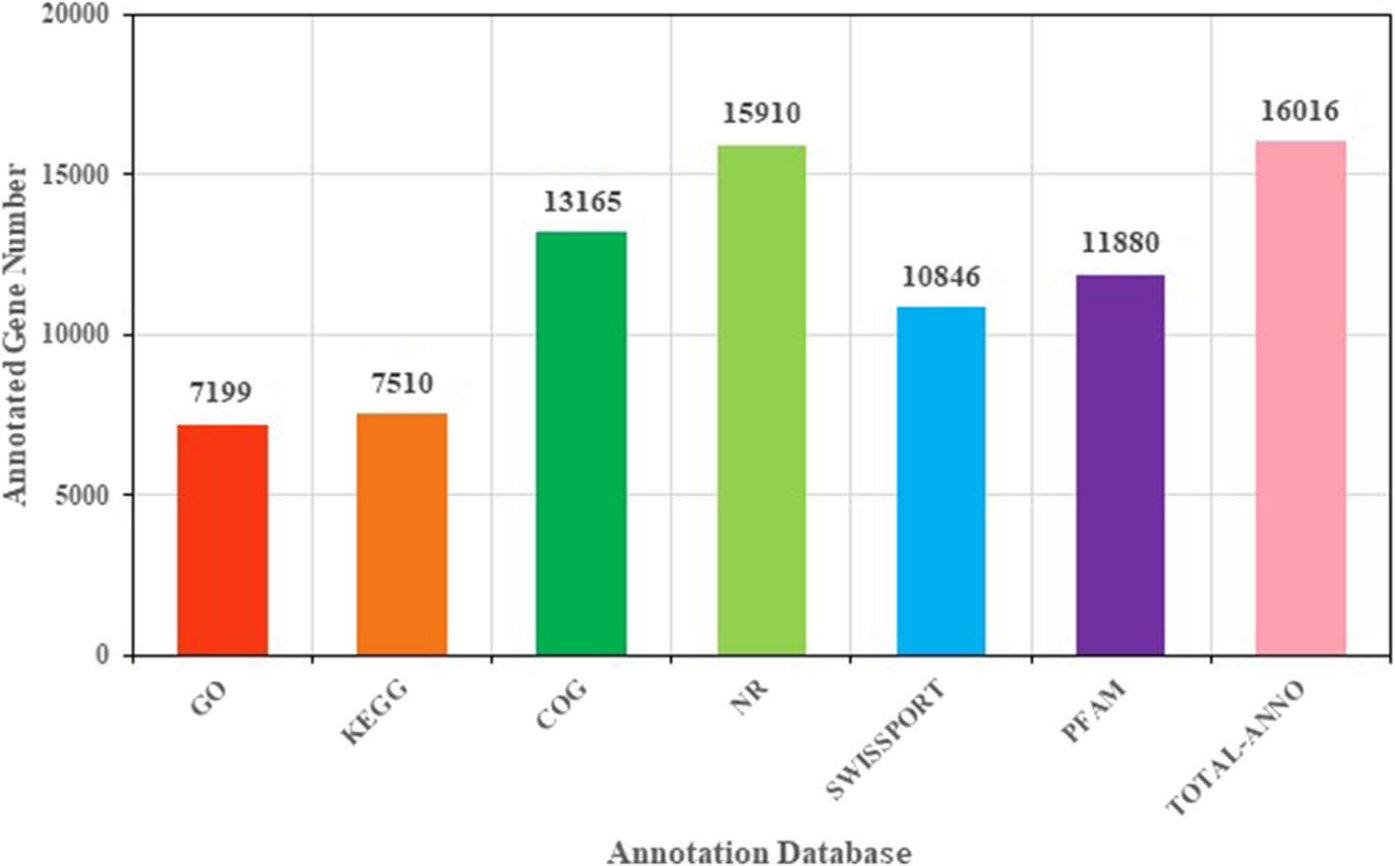
Functional annotation of genes to different databases

The unigenes were compared using six databases (NR, Swiss-Prot, Pfam, Eggnog, gene ontology [GO], and Kyoto Encyclopedia of Genes and Genomes [KEGG]). A total of 16,016 (0.73%) unigenes were annotated in this study through GO database matching (7,199; 33%), followed by KEGG (7,510; 34%), COG (13,165; 60%), NR (15,910; 73%), Swiss-Prot (10,846; 50%), and Pfam (11,880; 54%) databases (Fig. 2).

### Overview of *B. tabaci* differentially expressed genes (DEGs) in response to different treatments

**Fig. 3:**
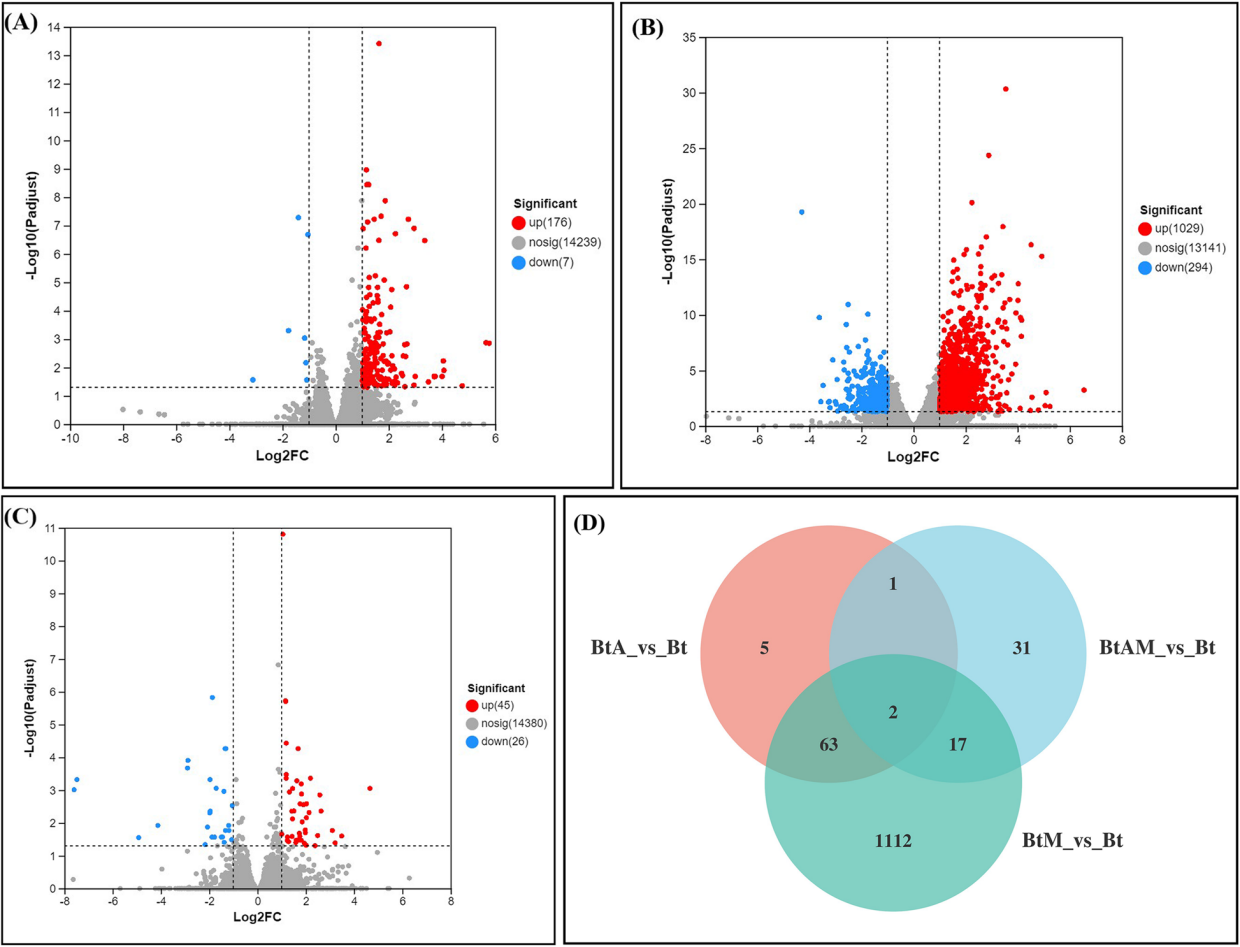
Differently expressed genes (DEGs) in B. tabaci treated with different treatments. **A** A. attenuatus versus control; **B** matrine versus control; **C** A. attenuatus + matrine versus control and **D** Venn diagram of the differentially expressed genes

Our results showed differential expression of 71 (69 upregulated and two downregulated) DEGs in whiteflies treated with *A. attenuatus* compared to whiteflies from the control treatment (BtA-Vs-Bt) (Fig. 3A), among which the majority of genes had Log_2_FC values within the range of 1 to 2 (69 genes; 97.18%). The number of genes with Log_2_FC values <-1 was 2 (2.81%) (Additional file 2A). A total of 1,194 (936 upregulated and 258 downregulated) DEGs were observed in whiteflies treated with matrine compared with those from the control treatment group (BtM-Vs-Bt) (Fig. 3B). Among these genes, the majority had Log_2_FC values in the range of 1 to 2 (790 genes; 66.16%). There were 146 genes (12.22%) with Log_2_FC values >2, and 258 genes (21.60%) with Log_2_FC values <-1 were observed (Additional file 2B). Our findings revealed differential expression of 51 (35 upregulated and 16 downregulated) DEGs in whiteflies treated with *A. attenuatus* + matrine compared to whiteflies from the control treatment (BtAM-Vs-Bt) (Fig. 3C), among which the majority of genes had Log_2_FC values in the range of 1 to 2 (35 genes; 68.62%), whereas 16 genes (31.37%) with Log_2_Ratio values <-1 were observed (Additional file 2C).

The cluster analysis of DEGs showed that the Log_2_FC values of most genes in the treatment groups were positive, which indicated that these genes were upregulated compared with those in the control groups (Additional file 2D).

### DEGs following treatment with *A. attenuatus*

Among the 69 genes that were upregulated in whiteflies treated with *A. attenuatus* compared to whiteflies from the control treatment, 19 were classified as orphan genes because they did not show any homology to known proteins. Another major category of upregulated DEGs in whiteflies treated with *A. attenuatus* consisted of five cuticle proteins (*Bta07904*, cuticle protein; *Bta07415*, cuticle protein; *Bta00253*, cuticular protein 73D; *Bta07899*, cuticular protein BJ; *Bta09581*, cuticle protein). A group of upregulated DEGs in whiteflies treated with *A. attenuatus* was fatty acyl CoA reductase I consisting of three genes (*Bta07543, Bta01237, Bta14907*) (Additional file 2A). The main upregulated DEGs involved in *B. tabaci* response to *A. attenuatus* were vitellogenin, cuticle protein, fatty acyl-CoA reductase 1, and catalase (Table 1).

**Table 1:** Top differentially expressed genes (DEGs) in whitefly treated with *A. attenuates* (A), matrine (B), and *A. attenuates* + matrine (C)

**A**						
Gene ID	Annotation	Control	A	FC(A/CK)	Log_2_FC(A/CK)	P-adjust
Bta09559	Unknown protein	0.88	5.44	3.31	1.72	3.5831E-08
Bta04635	Unknown protein	0.38	2.70	3.23	1.69	1.46223E-07
Bta08790	Unknown protein	0.40	3.89	3.11	1.63	7.81903E-07
Bta11224	Unknown protein	241.30	1103.24	2.95	1.56	2.37126E-07
Bta12487	Unknown protein	176.79	631.35	2.80	1.49	1.45403E-08
Bta14008	Unknown protein	0.79	4.56	2.76	1.47	1.69005E-05
Bta07853	Vitellogenin	62.29	180.34	2.75	1.46	3.84319E-14
Bta07904	Cuticle protein	182.01	556.14	2.63	1.40	4.34525E-08
Bta10132	Matrix metalloproteinase-14	14.02	57.39	2.61	1.39	2.29456E-05
Bta05091	Galactose oxidase	2.27	4.40	2.54	1.35	6.93277E-06
Bta01237	Fatty acyl-CoA reductase 1	10.15	40.23	2.50	1.32	9.29332E-05
Bta07415	Cuticle protein	21.09	58.77	2.49	1.32	3.34114E-07
Bta00253	Cuticular protein 73D	2.69	7.18	2.35	1.23	1.50461E-05
Bta13094	rRNA N-glycosidase	1.66	6.22	2.33	1.22	0.000620838
Bta13103	rRNA N-glycosidase	1.82	5.76	2.34	1.23	0.000146606
Bta03997	Xaa-Pro aminopeptidase 1	3.43	7.25	2.32	1.21	4.16415E-05
Bta14060	Mitochondrial phosphate carrier protein	144.89	378.82	2.32	1.21	6.12578E-06
Bta08493	Catalase	2.73	7.58	2.31	1.21	4.83081E-05
Bta01761	von Willebrand factor C domain-containing protein 2-like	6.39	2.16	0.43	-1.21	4.47058E-08
Bta05757	CTLMA2	63.20	15.56	0.44	-1.18	0.000570512
**B**						
Gene_id	Gene description	CK	B	FC(B/CK)	Log_2_FC(B/CK)	Padjust
Bta04297	Alpha-glucosidase	7.01	176.55	16.12	4.01	9.29576E-16
Bta03111	Cytochrome P450, putative	0.39	7.04	14.36	3.84	2.57545E-17
Bta08790	Unknown protein	0.40	5.15	10.73	3.42	5.64816E-14
Bta13768	Unknown protein	1.45	13.33	10.18	3.35	2.00273E-31
Bta13232	Unknown protein	3.87	50.47	9.89	3.31	3.84221E-10
Bta09175	Lupus la ribonucleoprotein, putative	0.99	14.25	9.88	3.30	1.74723E-10
Bta10684	Beta-1,3-glucan-binding protein	0.22	2.58	9.62	3.27	1.17294E-11
Bta08267	Protein takeout	8.41	89.02	8.97	3.16	4.72212E-12
Bta03997	Xaa-Pro aminopeptidase 1	3.43	15.40	8.85	3.15	1.0929E-18
Bta13640	Chemosensory protein	116.14	1059.78	8.21	3.04	9.46442E-12
Bta00818	Protein LEA-1, isoform m	3.52	22.28	6.67	2.740	1.50553E-15
Bta01237	Fatty acyl-CoA reductase 1	10.15	50.51	4.90	2.29	4.76606E-09
Bta09536	CytochromeP450	5.28	22.15	4.80	2.26	7.2037E-14
Bta15123	Unknown protein	2822.04	319.98	0.21	-2.23	9.57571E-06
Bta07683	Unknown protein	1824.55	237.91	0.21	-2.28	8.65592E-08
Bta09678	Unknown protein	7.63	2.09	0.21	-2.29	7.08689E-05
Bta05743	ACYPI009429 protein	230.36	26.89	0.20	-2.29	1.80388E-06
Bta01566	Unknown protein	4149.56	581.19	0.20	-2.33	1.19697E-11
Bta08284	Cuticle protein 6	166.64	22.46	0.20	-2.35	7.53313E-10
Bta05341	Unknown protein	11.80	0.64	0.19	-2.39	0.000175318
Bta13899	Cytochrome P450	6.64	0.58	0.17	-2.54	8.77223E-07
Bta05757	CTLMA2	63.20	3.84	0.12	-3.05	1.9712E-10
Bta08486	Unknown protein	266.06	10.61	0.07	-3.78	6.61647E-20
**C**						
Gene_id	Gene description	CK	C	FC(C/CK)	Log_2_FC(C/CK)	Padjust
Bta12247	Unknown protein	4.59	13.64	2.65	1.41	0.000375466
Bta03375	Transmembrane protease serine 3	1.44	3.01	2.50	1.32	5.22203E-05
Bta15139	Unknown protein	51.26	226.78	2.50	1.32	0.002771036
Bta09564	Cuticle protein	98.56	254.20	2.44	1.29	0.000717401
Bta02136	Histone H1.1	0.68	2.80	2.43	1.28	0.00427513
Bta03900	Tubulin alpha-3 chain	317.97	950.16	2.40	1.26	0.003177061
Bta14754	Unknown protein	362.76	866.40	2.39	1.26	0.001552716
Bta02475	Dopamine beta-hydroxylase, putative	2041.66	6107.13	2.36	1.24	0.003177061
Bta06515	ATP-dependent DNA helicase PIF1	10.07	22.42	2.35	1.24	0.000510084
Bta08328	Unknown protein	13.61	43.41	2.34	1.23	0.006081865
Bta13272	63 kDa sperm flagellar membrane protein	8.67	2.36	0.46	-1.12	5.22203E-05
Bta11374	CG16798	34.23	9.17	0.45	-1.14	5.22203E-05
Bta04938	Pericardin	0.67	0.11	0.45	-1.16	0.013872136
Bta09690	Unknown protein	7.92	1.28	0.43	-1.21	0.005761438
Bta04161	Unknown protein	5.99	0.97	0.43	-1.22	0.004522648
Bta13466	Peroxidase	4.98	1.01	0.42	-1.25	0.000868854
Bta06515	ATP-dependent DNA helicase PIF1	10.07	22.42	2.35	1.24	0.000510084
Bta01616	Protein msta, isoform A	4.71	1.06	0.36	-1.48	1.29694E-06
Bta05871	von Willebrand factor A domain-containing protein 2	5.23	0.47	0.35	-1.51	0.000230769
Bta10028	Unknown protein	2.06	0.18	0.35	-1.51	0.000230769

Only two downregulated DEGs were found in whiteflies treated with *A. attenuatus* compared to whiteflies from the control treatment. The two downregulated DEGs included von Willebrand factor C domain-containing protein 2-like (*Bta01761*) and CTLMA2 (*Bta05757*) (Table 1).

### DEGs following treatment with matrine

Numerous upregulated genes (936) were observed in whiteflies treated with matrine compared with those from the control treatment. Among the 936 upregulated DEGs, five major categories were identified, namely 245 orphan genes, 21 cathepsins, 19 cuticle proteins, 17 cytochrome P450, and five fatty acyl-CoA reductase 1 genes. Two categories, orphan genes and cuticle proteins, were also differentially regulated in whiteflies treated with *A. attenuatus* compared to those from the control treatment. Only 18 out of 245 orphan genes that were differentially expressed between whiteflies treated with matrine were similar to those differentially expressed in whiteflies treated with *A. attenuatus.* Furthermore, four cuticle protein genes (*Bta07904, Bta00253, Bta07899,* and *Bta09581*) were similar to those differentially expressed in whiteflies treated with *A. attenuatus* (Additional file 2 B)*.* The major upregulated DEGs involved in *B. tabaci* response to matrine were alpha-glucosidase I, cytochrome P450, vitellogenin, fatty acyl-CoA reductase 1, protein LEA isoform M, and cathepsin F-like protease, which were also upregulated with Log_2_FC values in the range of 2–3 (Table 1).

Among the 258 genes downregulated in whiteflies treated with *matrine* compared to whiteflies from the control treatment, 60 were classified as orphan genes. Another category of downregulated DEGs in whiteflies treated with matrine consisted of three cuticle proteins (*Bta08284*, cuticle protein 6; *Bta14107*, cuticle protein 1; *and Bta01013*, cuticular protein, putative). One group of downregulated DEGs in whiteflies treated with matrine was cytochrome P450, which consisted of three genes (*Bta13899, Bta11266,* and *Bta06109*). The two downregulated DEGs involved in the response of *B. tabaci* to matrine were von Willebrand factor C domain-containing protein 2-like (*Bta01761*) and CTLMA2 (*Bta05757*) (Table 2).

**Table 2:** Primers used in the study

Primer	Primer Sequence (5’-3’)
XM_019059872 F	GCGGCTCACTTGAAAGTTTCGTC
XM_019059872 R	GACTCCGTCTTTCGCATCGTCAT
XM_019040592 F	CACGTCTTGGGGCGAGAAAGG
XM_019040592 R	CGCGACGCCACTGACCACTTAC
XM_019057203 F	TGCTGGGAGGAAACATGGAA
XM_019057203 R	AACGATGGGCACCGCTATT
XM_019041810 F	CGGCAGCCCAGTATCACAA
XM_019041810 R	CGGCGTCTGTCTCCTCTTTG
XM_019053324 F	CCGAGGGATGACGCACGATA
XM_019053324 R	AGGGTCCTGGGCTTGGGTTT
XM_019044430 F	GAGCATACCGAGGCTTCCATC
XM_019044430 R	TCCAACTGCTGCGGAGGTTA
XM_019055894 F	ATCGCCCATCTTCACCTCCG
XM_019055894 R	TTTCATCCGACTTCCCCGTAG
XM_019055894 F	TGATACTCAGGGCCGCTGTT
XM_019055894 R	TGACGCTCTGTCGTGCTCTTTA
XM_019047326 F	CTTTCAGCCATTTGTCCTTTACG
XM_019047326 R	AAGTCCAAACCTCTGGTTTCCTT
β-actin F	TCTTCCAGCCATCCTTCTTG

### DEGs following treatment with *A. attenuatus +* matrine

In total, 35 DEGs were upregulated in whiteflies treated with *A. attenuatus +* matrine compared to those from the control treatment. Among the 35 upregulated DEGs, three major categories were identified: 12 orphan genes, three cuticle proteins, and two histone H1 genes. As described above, cuticle proteins were also differentially regulated in whiteflies treated individually with *A. attenuatus* and matrine compared to whiteflies from the control treatment. Only one of three cuticle protein genes (*Bta09545*) was similar to those differentially expressed in whiteflies treated with matrine*.* Furthermore, two upregulated DEGs (*Bta09545:* Protein LEA-1and *Bta05911*: Cathepsin F-like protease) were also differentially regulated in whiteflies treated with individual treatments of *A. attenuatus* and matrine compared to whiteflies from the control treatment (Additional file 2C). The major upregulated DEGs involved in *B. tabaci* response to *A. attenuatus* + matrine were transmembrane protease serine 3, cuticle protein, tubulin alpha-3 chain, and histone H1.1 (Table 2).

Among the 16 genes that were downregulated in whiteflies treated with *A. attenuatus +* matrine compared to whiteflies from the control treatment, four were classified as orphan genes. The twodownregulated DEGs involved in the response of *B. tabaci* to matrine were von Willebrand factor C domain-containing protein 2-like (*Bta01761*) and CTLMA2 (*Bta05757*) (Table 2).

### Common DEGs between whiteflies treated with individual treatments of *A. attenuatus* and matrine

Of the 72 and 1,194 DEGs found in whiteflies treated with individual treatment of *A. attenuatus* and matrine, respectively, there were 65 common genes that were found to be differentially expressed in both treatments. These common DEGs included several major categories: 1) 18 orphan genes; 2) four cuticle proteins (*Bta07904, Bta00253, Bta09581,* and *Bta07899*); 3) three cathepsins (*Bta02553* and *Bta01771*, cathepsin B; *Bta05911,* cathepsin F-like proteases); 4) three fatty acyl-CoA reductase 1 (*Bta01237, Bta07543,*and *Bta14907*); and 5) two glucose dehydrogenase genes (*Bta11629; Bta10911*) (Additional file 2D).

### Common DEGs between whiteflies treated with individual and combined treatments of *A. attenuatus* and matrine

Of the 72, 1,194, and 51 DEGs found in whiteflies treated with individual or combined treatments of *A. attenuatus* and matrine, respectively, two genes (one protein LEA-1 isoform gene (Bta00818) and 2) cathepsin F-like protease (*Bta05911*)) were differentially expressed between all three treatments (Additional file 2E).

### Correlation between DEGs and mortality of whiteflies treated with individual and combined treatments of *A. attenuatus* and matrine

The correlation analysis between DEGs and mortality of whiteflies treated with individual and combined treatments of *A. attenuatus* and matrine revelaed a negative correlation (-0.768) between both variables.

### KEGG pathway analysis

**Fig. 4:**
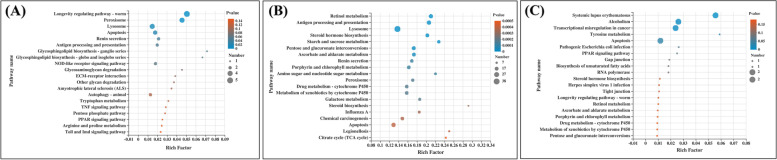
Most enriched Kyoto Encyclopedia of Genes and Genomes (KEGG) pathways in B. tabaci treated with different treatments. **A** A. attenuatus versus control; **B** matrine versus control; and **C** A. attenuatus + matrine versus control

KEGG pathway enrichment analysis of DEGs was performed to identify the potential pathways that were upregulated/ downregulated in whiteflies treated with different treatments (*A. attenuatus*; matrine; *A. attenuatus* + matrine). In whiteflies treated with *A. attenuatus*, the most enriched pathways were “Longevity regulating pathway - worm,” “Renin secretion,” and “Antigen processing and presentation” of Organismal Systems; “Peroxisome,” “Lysosome,” and “Apoptosis” of Cellular Processes, and “Glycosphingolipid biosynthesis - ganglio series, Glycosphingolipid biosynthesis - globo and isoglobo series of Metabolism (Fig. 4A, Additional file 3). Furthermore, 80% (57 of 71) of the total number of upregulated DEGs in whiteflies treated with *A. attenuatus* were annotated to KEGG pathways. The three major categories of pathways most represented as upregulated in whiteflies treated with *A. attenuatus* were 1) metabolism, 2) organismal systems, and 3) cellular processes (Additional file 4A). KEGG analysis was not performed for downregulated DEGs in whiteflies treated with *A. attenuatus* because two DEGs were downregulated.

In whiteflies treated with matrine, 47 KEGG pathways were significantly enriched. Within the top 10 enriched pathways, most of them were associated with the Metabolism category, including the “Retinol metabolism,” “Antigen processing and presentation,” “Starch and sucrose metabolism,” “Pentose and glucuronate interconversions,” “Ascorbate and aldarate metabolism,” “Porphyrin and chlorophyll metabolism,” and “Amino sugar and nucleotide sugar metabolism” (Fig. 4B, Additional file 3). Furthermore, 90% (1,077 of 1,194) DEGs were annotated to KEGG pathways among the upregulated DEGs, and accounted for four main categories: 1) metabolism, 2) human diseases, 3) organismal systems, and 4) cellular processes (Additional file 4B). The downregulated DEGs in whiteflies treated with matrine were associated with the following main categories: 1) organismal system, 2) metabolism, 3) environmental information processing, and 4) human diseases (Additional file 4C).

In whiteflies treated with *A. attenuatus* + matrine, the most enriched pathways were “Systemic lupus erythematosus” of the Immune disease subcategory and “Apoptosis” of the Cell growth and death subcategory (Figure 4C, Additional file 3). In total, 62% (32 out of 51) of the total number of upregulated DEGs in whiteflies treated with *A. attenuatus* + matrine were annotated to KEGG pathways. The three major categories of pathways most represented as upregulated in whiteflies treated with *A. attenuatus* were metabolism, human diseases, and cellular processes (Additional file 4D).

### Quantitative real-time polymerase chain reaction (qRT- PCR) validation of DEGs

**Fig. 5:**
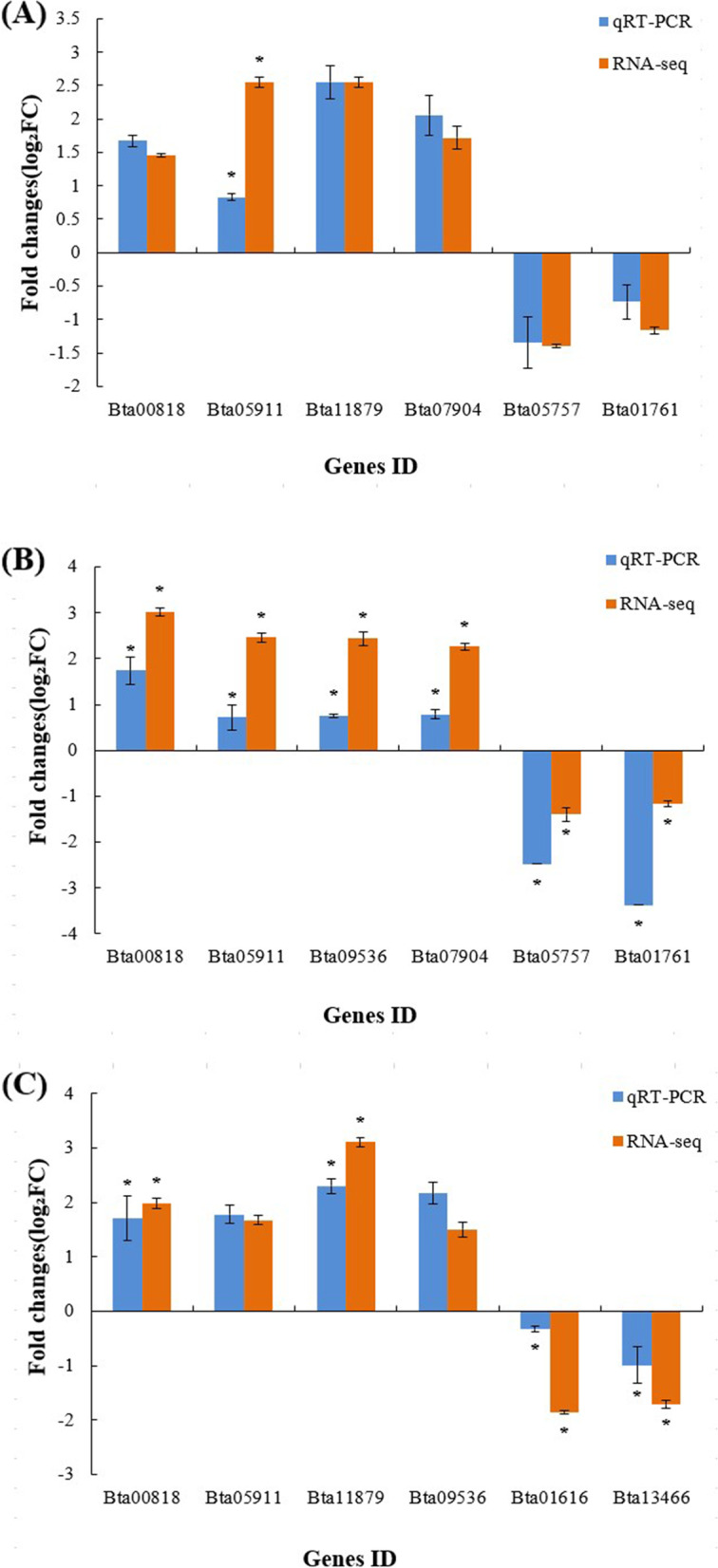
qRT-PCR validation of differently expressed genes (DEGs) expressed by *B. tabaci* in response to different treatments. (**A**) *A. attenuatus*; (**B**) matrine; and (**C**) *A. attenuatus* + matrine. Error bars represent standard deviation of the mean of three biological replicates

To validate the digital gene expression map (DGE) data, the transcript levels of eight randomly selected genes (six upregulated and two downregulated) were quantified by qRT-PCR, using RNA extracted from three independent biological replicates. In all three biological replicates, ten genes showed concordant changes between the DGE and qRT-PCR data (Figure 5 and Additional file 5), suggesting that the DGE data were reliable.

## Discussion

*Akanthomyces attenuatus* Zare & Gams is a well-known pathogen of insect pests, including whiteflies, aphids, and thrips. Recently, many studies have investigated the joint application of pathogenic insect fungi and natural enemies, synthetic oils, bacterial metabolite avermectins, and other biopesticides. Our recent studies on the combined application of *A. attenuatus* and matrine (a botanical insecticide) against *B. tabaci* have shown promising results. To date, no study has been conducted to elucidate the immune response of *B. tabaci* to the combined application of *A. attenuatus* and matrine at the molecular level. In the present study, we obtained a transcriptome dataset containing 35,895 unigenes of *B. tabaci* using RNA-Seq. The analysis of DEGs involved in the immune response to single or joint treatments of *A. attenuatus* and matrine revealed the expression of 5, 54, and 0 immunity-related genes for BtA, BtM, and BtAM, respectively. Most immunity-related genes are also involved in NOD-like receptor signaling, Toll and Imd signaling, Fc gamma R-mediated phagocytosis, and antigen processing and presentation pathways. Our findings differ from those of Xia et al. [21], who examined the expression of *B. tabaci* genes in response to infection with *Beauveria bassiana*. Their findings revealed that 654 and 1,681 genes were differentially expressed at 48 and 72 h post-infection, respectively, compared to the control. Chen et al. [26] also observed a lower number of DEGs in *Megalurothrips usitatus* post-*A. attenuatus* infection when compared to infection with *Beauveria brongniartii*. The lower number of DEGs and zero expression of immunity-related genes by *B. tabaci* treated with combined treatments of *A. attenuatus* and matrine can be related to the disruption of the defense/immune system of the host [7]. The DEGs were grouped according to their functional roles.

### Differential expression of metabolism and biogenesis-related genes

Insects respond to external stress by increasing carbohydrate metabolism and energy production [27]. Major differences were observed within the metabolism category between whiteflies subjected to different treatments. In total, 19 metabolic pathway genes (all upregulated) were found to be differentially expressed in whiteflies treated with *A. attenuatus* compared with 359 (324 upregulated and 35 downregulated) metabolic pathway DEGs in whiteflies treated with matrine and 12 metabolic pathway genes (all upregulated) in whiteflies treated with *A. attenuatus* + matrine. We observed an overall lower differential expression of metabolism-related genes (after treatment with *A. attenuatus* or matrine + *A. attenuatus*), which may play an important role in the complex interactions between B. tabaci and *A. attenuatus*. *B.* tabaci may reduce general metabolism and conserve energy to fight fungal infections with alternative immune responses or reproductive strategies [28–31]; however, this global downregulation could be regulated by fungal effectors that suppress host genetic information processing and metabolism.

Membrane-bound acyl-lipid desaturases and membrane-bound acyl-CoA desaturases are two types of membrane-bound fatty acid desaturases. Insect acyl-CoA desaturases play a critical role in semiochemical biosynthesis and sensing, cold tolerance, defensive fatty acid production, lipid metabolism, feeding behavior, and larval development [32–36]. Our results showed the upregulation of the acyl-CoA desaturase gene (*Bta11879*) in response to individual or combined treatments with pathogens and botanicals. The higher expression levels of the acyl-CoA desaturase gene in *B. tabaci* following different treatments suggest the acceleration of lipid metabolism to produce the necessary energy required for the induction of a strong immune response [37].

Cytochrome P450 monooxygenases are enzymes found throughout the animal kingdom and are mainly involved in metabolism [38]. Toxins can trigger cytochrome P450 production in the diet, which makes this adaptation noteworthy [39]. In addition, cytochrome P450 activities are known to be involved in insecticide metabolism, resulting in molecular activation or, more broadly, detoxification [40, 41]. Cytochrome P450, which is responsible for the specificity of the reaction, is the primary source of proteins in each of these enzymatic systems. In this study, cytochrome P450 was upregulated in response to individual treatments with matrine and *A. attenuatus +* matrine, confirming the increased detoxification response of *B. tabaci* to the different treatments.

### Differential expression of signaling-related genes

Among the genes annotated to signal transduction pathways, four upregulated DEGs were observed in whiteflies treated with *A. attenuatus*. In whiteflies treated with matrine, 123 DEGs (89 upregulated and 34 downregulated) were associated with signal transduction pathways, whereas only one upregulated DEG was annotated to signal transduction pathways.

In insects, FAR genes involved in sex pheromone production (pheromone-gland-specific fatty acyl reductase) form a distinct clade with significant changes in gene structure, fatty acyl substrate specificity, and selectivity [42]. Fatty acyl-CoA reductase (FAR) is the key enzyme responsible for the biosynthesis of fatty alcohols and can catalyze the transformation of fatty acids to fatty alcohols and NADPH [43, 44]. Our results showed upregulation of the fatty acyl-CoA reductase 1 gene (*Bta11879*) in response to individual treatments with pathogens and botanicals. These findings suggest that fatty acid metabolism may be involved in the detoxification of pathogens and botanicals in whiteflies. Upregulated expression of fatty acyl-CoA reductase 1 gene may produce fatty alcohol and wax-ester in the insect epicuticle [45].

Histone H1 is one of the important components of eukaryotic chromatin. This protein is known to play a key role in the chromatin folding into higher-order structure (Allan et al. 1980). It was shown to be a nonspecific repressor of gene expression [46]. Besides, histone HI appears to compete with specific gene activators for regulatory sites of tissue-specific genes, thus determining their ability for further transcription [46]. Moreover, HI is a member of the regulatory cascade involved in the action of nuclear receptors which leads to selective activation of chromatin. Several kinds of indirect evidence suggest that molecular variations in these proteins are likely to have pleiotropic effects, including effects on quantitative traits (growth, fecundity etc.) [47]. The upregulation of Histone H1 gene in whitefly nymphs treated with matrine+*A. attenuatus* might be related to their effort to cope with external stress through higher transcription or growth activities.

### Differential Expression of immunity-related genes

The analysis of DEGs that may participate in the immune response to single or combined treatments of *A. attenuatus* and matrine (BtA, BtM, and BtAM) revealed some interesting findings. Among whiteflies treated with *A. attenuatus*, 5 immunity-related (five upregulated, zero downregulated) DEGs were observed. These DEGs were mainly involved in the NOD-like receptor signaling pathway (map04621), the Toll and Imd signaling pathway (map04624), and the antigen processing and presentation pathway (04612). In whiteflies treated with matrine, 54 immunity-related DEGs (51 upregulated and three downregulated) were observed. The majority of immunity DEGs were annotated to the NOD-like receptor signaling pathway (map04621), Toll and Imd signaling pathway (map04624), Fc gamma R-mediated phagocytosis (map 04666), and antigen processing and presentation pathway (04612). In whiteflies treated with *A. attenuatus* + matrine, no immunity-related gene was expressed, which confirmed the complete disruption of the *B. tabaci* immune response by the combined application of *A. attenuatus* and matrine Vitellogenins are insect proteins that provide nutrients for yolk formation [48, 49]. Many studies have revealed that vitellogenins play an essential role in the immune system of different insect species [48, 50, 51]. In this study, it was also found that the expression of some vitellogenins was upregulated after treatment with pathogens (*A. attenuatus*), botanicals (matrine), and their combination (*A. attenuatus +* matrine), thereby confirming the role of vitellogenins in *B. tabaci* defense against different treatments.

In cysteine proteases, cysteine residues modulate catalytic processes [27]. These endopeptidases can regulate digestion, embryonic vitellin degradation, and metamorphosis in insects [28, 29]. Cysteine cathepsins (B, F, L, and O) are the most important cysteine proteases observed in insects, and are well known for their endopeptidase activity [30]. Recent studies have shown that cathepsin F-like enzymes are, quantitatively, the most important cysteine proteases in insects [31]. Miyaji et al. [32] showed that CathF was involved in the maintenance of endogenous cysteine protease activity in the larval hemolymph of *Manduca sexta*. Our results showed the upregulation of cathepsin F-like protease gene (*Bta05911*) in response to individual or combined treatments of pathogen and botanical. The upregulated expression of cathepsin F-like protease genes in response to different treatments shows the importance of CathF in the immune defense of *B. tabaci* through antigen processing and presentation [32].

The insect cuticle is the first barrier against pathogen infection and maintains the form and movement of the insects [52]. The cuticle is a protective envelope that can completely enclose and protect insect larvae from the external environment [32]. Cuticles mainly comprise chitin, lipids, catecholamines, minerals, and proteins [33]. Cuticle proteins are induced to improve the stability of the cuticle required for insect survival under external stress [52]. One of the routes of insecticide resistance is cuticle thickening [34]. Environmental stress enhances the expression of cuticle proteins in *Leptinotarsa decemlineata* in response to stress, allowing the insects to adapt to new or changing settings [35]. Silva et al. [36] found that cuticle protein genes of aphids were upregulated following insecticide application. In this study, the expression of cuticle protein was upregulated in response to individual treatments of pathogen (*A. attenuatus*) and botanical (matrine), whereas in the case of *A. attenuatus +* matrine, there was no or minimal expression of cuticle protein genes, which indicated disruption of the *B. tabaci* immune process.

Late embryogenesis abundant (LEA) proteins are involved in insect response to environmental stress and thus act as water deficiency stress protectants [53]. The LEA proteins act as a chemical chaperone for trehalose (the most abundant hemolymph sugar in insects) and may preserve cell components from desiccation by replacing water or forming glass [54]. Kikawada et al. [38] reported that LEA protein 1 was accumulated in response to desiccation or NaCl treatment in *Polypedilum vanderplanki* larvae. Our results showed the upregulation of the LEA protein-1 gene (Bta000818) in response to individual or combined treatments of pathogen and botanical. After different treatments, the upregulation of the LEA protein-1 gene by *B. tabaci* confirmed a strong immunological/detoxification response through trehalose accumulation to survive drying in response to infection [39].

C-type lectins (CTLs) are involved in the binding site of glycans in direct coordination with lectin-bound Ca^2+^ [55]. CTLs are divided into two classes based on their binding preference. CTLs with the EPN sequence at the calcium-binding site prefer mannose-type sugars (fucose, glucose), whereas CTLs with the QPD sequence prefer galactose-type sugars [56]. The CTL proteins CTL4 and CTLMA2 have been reported to influence the insect immune response during microbial infection by stabilizing phenol oxidase activity and reducing the downstream melanization response [57, 58]. Our results showed that the CLTMA2 gene (Bta05757) is downregulated in response to individual or combined treatments with pathogens and botanicals. Lower expression levels of CLTMA2 in *B. tabaci* following different treatments could have resulted in a weaker immune response and increased melanization, implying susceptible whitefly individuals [59].

Single von Willebrand factor C-domain proteins (SVWCs) are single-domain von Willebrand factor type C proteins known primarily from arthropods [60, 61]. Kleinjung et al. [62] discovered the first SVWC in *Drosophila melanogaster*. SVWC proteins are involved in response to environmental stressors, changes in nutritional status, and microbial infections [63]. Our results showed downregulation of the von Willebrand factor type C protein-2 gene (*Bta01761*) in response to individual or combined treatments of pathogens and botanicals. The lower expression levels of the von Willebrand factor type C protein-2 gene in *B. tabaci* following different treatments resulted in a reduced immune response during pathogen invasion through reduced phagocytosis and antimicrobial responses [55, 56].

A large amount of energy is normally required for a rapid and effective immune response [64]. In insects, energy is derived from carbohydrate sources such as glycogen and trehalose. These two stored sugars are enzymatically converted into glucose, which is then used in the production of high-energy ATP through glycolysis and the tricarboxylic acid (TCA) cycle [65]. This metabolic switch is clearly essential for antimicrobial responses, because starvation or blocking the signal transduction for metabolic reprogramming has been found to decrease the immune response against pathogens [66]. Our results further support the possible significance metabolic switching in antimicrobial responses as the lower differential expression of metabolic pathways related genes in whiteflies treated with *A. attenuatus* and matrine+*A. attenuatus* treatments ultimately led to the lower expression of immunity related genes. In short, reduced/disrupted metabolic response might have led to the lesser number of diferentially expressed genes in whiteflies exposed to matrine+*A. attenuatus* when compared to the remain treatments.

## Conclusion

In summary, we investigated the complex interactions between the whitefly and individual or combined treatments of *A. attenuatus* and matrine using RNA sequencing. Our data indicated that genes such as cytochrome P450, cuticle protein, vitellogenin, cysteine proteases, late embryogenesis abundant (LEA) proteins, desaturases, CTLMA2, and von Willebrand factor type C protein-2 were mainly involved in whitefly defense responses against *A. attenuatus* and matrine. The results also revealed that AMPK signaling, apoptosis, and drug metabolism pathways are likely involved in whitefly defense responses against *A. attenuatus* and matrine infection. To the best of our knowledge, this is the first report on the molecular interactions occurring between whiteflies and individual or combined treatments of *A. attenuatus* and matrine. These results will further improve our knowledge of the infection mechanism and complex biochemical processes involved in the synergistic action of *A. attenuatus* and matrine against *B. tabaci*.

## Methods

### Insect cultures

Whitefly cultures were reared on cotton plants according to the method described by Wang et al. [3] at the Engineering Research Centre of Biological Control, Ministry of Education, South China Agricultural University, under standard laboratory conditions (26 °C, 70% relative humidity (RH), and 12:12 h light/dark photoperiod).

### Preparation of fungal inoculum and whitefly treatment

*Akanthomyces attenuatus* isolate SCAUDCL53 (deposited at the Key Laboratory of Biopesticides Innovation and Application of Guangdong Province, South China Agricultural University, Guangzhou, P.R. China) was cultured according to the method described by Ali et al. [67] to calibrate the basal fungal suspension of 1×10^6^ conidia/ml.

Matrine (98% purity) was purchased from Guangdong New Scene Bioengineering Company Ltd., Yangjiang, P.R. China, and the basal concentration of matrine (1.0 mg/mL) was prepared following the methodology of Wu et al. [17].

Cotton leaves having 2^nd^ instar *B. tabaci* nymphs were individually dipped in *A. attenuatus* fungal suspension (1×0^6^ conidia/ml), matrine (1.0 mg/L), and *A. attenuatus* and matrine (1×0^6^ conidia/ml + 1.0 mg/L) for 10 seconds. The leaves were air dried before being placed in a 9 cm agar medium with 20% agar to maintain moisture. *Bemisia tabaci* dipped in 0.05% Tween 80 served as control. Each treatment consisted of four cotton leaves with 100 *B. tabaci* nymphs per leaf. The experimental setup was maintained at 25 °C, 85% RH, and a 16:8 h (light/dark photoperiod). The entire experiment was repeated three times (with a fresh batch of insects and new conidial suspension). The mortality of the insects was tracked daily for 5 d after treatment.

### RNA extraction for sequencing

The surviving *B. tabaci* nymphs (200 nymphs pooled per sample and three samples for each treatment considered as three replicates) from different treatments were collected in 1.5 mL Eppendorf tubes after approximately 3 d of treatment, frozen in liquid nitrogen, and stored at -80 °C. Total RNA was extracted using TRIzol reagent (Thermo Fisher Scientific, Germany), according to the manufacturer’s instructions (Invitrogen). The quality of the extracted RNA was determined using 1% agar-gel electrophoresis and a NanoDrop One ultraviolet spectrophotometer (Thermo Fisher Scientific, Germany), and the RNA integrity number (IN) value was calculated using an Agilent 2100 bioanalyzer (Agilenet, USA).

### Construction and sequencing of cDNA library

The mRNAs (post RNA extraction) were enriched with Oligo (dT) having magnetic beads treated with DNase I. The mixture was then supplemented with fragmentation buffer to prepare the mRNA templates for the sysnthesis of first cDNA chain whereas RNA enzyme, DNA polymerase I, and dNTPs were added to synthesize the second cDNA chain. AMPure XP beads were used for cDNA (double-stranded) purification followed by the selection of cDNA library through PCR enrichment.. The library was sequenced with Illumina sequencing platform.

### Assembly and annotation of the transcriptome

Raw reads were filtered and assembled using Cufflinks (http://cole-trapnell-lab.github.io/cufflinks/). The assembled sequences were removed from redundancy and spliced using TGICL to obtain the longest nonredundant unigene set. Further statistical analysis and quality control were performed using the unigene set. The unigene set obtained by de Novo and clustering was compared with the database using BLAST for functional annotation. The NR, Swiss-Prot, Pfam, EggNOG, GO, and KEGG databases were used for annotation. The annotation information of genes or transcripts could be comprehensively obtained, and the annotation of each database was determined. To understand the transcriptome database of the response of *B. tabaci* to *A****.*** *attenuatus* and matrine alone or in combination, 12 DGE marker libraries were constructed, including the transcription information of *B. tabaci* treated with *A****.*** *attenuatus* and matrine alone or in combination with a blank control group. The treatment groups were named as BtA-1, BtA-2, BtA-3, BtM-1, BtM-2, BtM-3, BtAM-1, BtAM-2, BtAM-3, Bt-1, Bt-2, and Bt-3. Table 2 summarizes the basic statistics of the 12 libraries.

### Digital gene expression profile analysis

Using the constructed cDNA library data, a DGE was used to identify the DEGs. We regarded genes with a false discovery rate ≤ 0.001 and |fold change| ≥ 2 as DEGs. Genes with similar expression patterns often exhibit similar functions. The R toolkit pheatMap software was used for cluster analysis of DEGs and experimental conditions. GO-seq was used for the GO enrichment analysis. The main difference between this method and the ordinary hypergeometric distribution is that the influence of gene length preference can be eliminated so that the GO term of true enrichment can be calculated more accurately. Combined pathways (KEGG) with the above pathways [68] and GO functional categories with significant enrichment of DEGs were obtained for further analysis.

### qRT-PCR verification of DGEs

Primer Premier 5 software (Premier Biosoft, San Francisco, USA) was used to create primers for the DEGs and a reference gene (Table 2). To validate the DEGs, 18 DEGs were selected for qRT-PCR analysis of the RNA samples used for sequencing. The expression analysis was performed using three biological replicates. SYBR Premix Ex Taq (Perfect Real Time, Takara Bio Inc., Japan) was used for the final reaction. The qRT-PCR system had a total volume of 20 μL, which included 0.4 μL of forward/reverse primers, 1 μL of 10-fold cDNA, 8.2 μL of nuclease-free Water, and 10 μL of 2×Perfect Start TM Green qPCR Super Mix. The qRT-PCR was conducted on a Bio-RAD iQ5 thermocycler with the following temperature settings: initial denaturation at 94 °C for 30 minutes, followed by 39 cycles of 94 °C for 5 seconds, 55 °C for 30 minutes, and 72 °C for 10 seconds, followed by a melt curve of 65-95 °C, incrementing 0.5 °C for 0.05 seconds. As described earlier, the 2^-ΔΔCt^ technique was used to calculate relative expression levels using β-actin as an internal control [21, 25, 26, 69, 70].

## Supplementary Information

**Additional file 1. **a. The number of Illumina raw and processed reads produced per RNA-Seq library from whiteflies (*Bemisia tabaci*) treated with individual or combined treatments of *Akanthomyces attenuatus* and matrine. b. Length distribution of *B. tabaci* unigenes. c. Correlation matrix analysis for multiple biological replicates obtained from RNA-Seq libraries prepared from whiteflies (*Bemisia tabaci*) treated with individual or combined treatments of *Akanthomyces attenuatus* and matrine.

**Additional file 2. **a. List of differentially expressed genes in whiteflies treated with *Akanthomyces attenuatus*. b. List of differentially expressed genes in whiteflies treated with matrine. c. List of differentially expressed genes in whiteflies treated with *Akanthomyces attenuatus* + matrine. d. Cluster analysis of differentially expressed genes in whiteflies treated with individual or combined treatments of *Akanthomyces attenuatus* and matrine. e. List of 66 common differentially expressed genes between whiteflies treated with individual treatments of *Akanthomyces attenuatus* and matrine (BtA and BtM). f. List of 66 common differentially expressed genes between whiteflies treated with individual and combined treatments of *Akanthomyces attenuatus* and matrine (BtA, BtM, and BtAM).

**Additional file 3. **The main Kyoto Encyclopedia of Genes and Genomes (KEGG) enrichment pathway in *B. tabaci* treated with *Akanthomyces attenuatus* matrine, *Akanthomyces attenuatus* + matrine, and controls.

**Additional file 4. **Summary of Kyoto Encyclopedia of Genes and Genomes (KEGG) reference pathways of whiteflies associated with different treatments. (A) *Akanthomyces attenuatus* upregulated pathways; (B) matrine upregulated pathways; (C) matrine downregulated pathways; and (D) *Akanthomyces attenuatus* + matrine upregulated pathways.

**Additional file 5.** Results of quantitative real time PCR.

## Data Availability

The sequencing data generated in this study were deposited in the NCBI SRA database. The raw datasets generated in this study are available at http://www.ncbi.nlm.nih.gov/bioproject/PRJNA797870 (NCBI accession number: PRJNA797870).
