# Supplementary figures and images for: Comparative transcriptome analysis reveals differences in gene expression in whitefly following individual or combined applications of Akanthomyces attenuatus (Zare & Gams) and matrine
Source: BMC Genomics. 2022 Dec 6;23:808. doi: 10.1186/s12864-022-09048-9 (PMC9727895; doi:10.1186/s12864-022-09048-9)

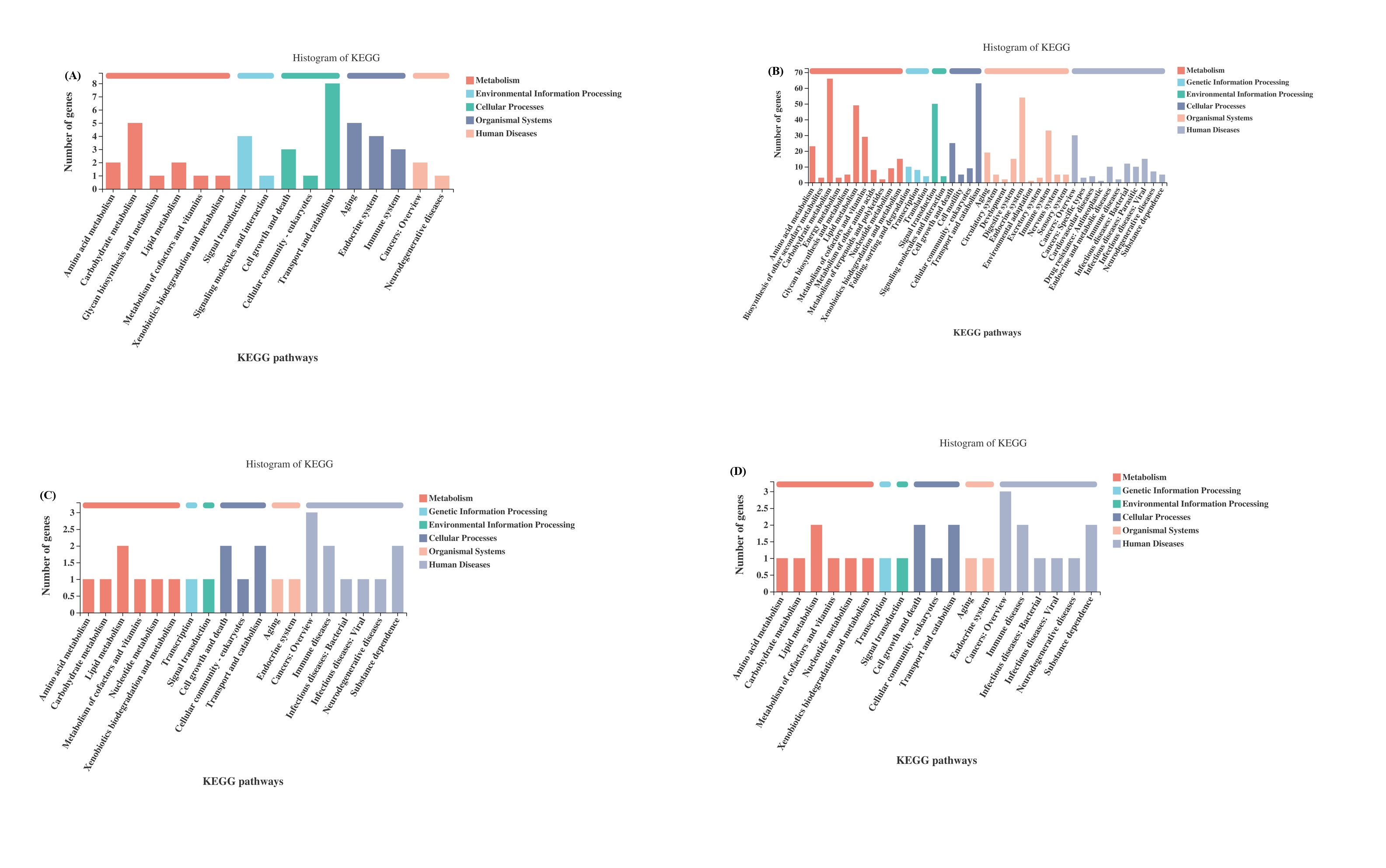

Supplement: Supplementary file 3 — Additional file 3. The main Kyoto Encyclopedia of Genes and Genomes (KEGG) enrichment pathway in B. tabaci treated with Akanthomyces attenuatus matrine, Akanthomyces attenuatus + matrine, and controls. [file 12864_2022_9048_MOESM3_ESM.jpg]
